# Susceptibility of *Aedes aegypti* to spinosad larvicide and space spray adulticides in Brazil

**DOI:** 10.1590/0074-02760240270

**Published:** 2025-07-11

**Authors:** Luciana dos Santos Dias, Ademir Jesus Martins, Cynara de Melo Rodovalho, Diogo Fernandes Bellinato, Tatiana Mingote Ferreira de Ázara, Aline Machado Rapello do Nascimento, Vincent Corbel, Maria de Lourdes da Graça Macoris, Maria Teresa Macoris Andrighetti, José Bento Pereira Lima

**Affiliations:** 1 Fundação Oswaldo Cruz-Fiocruz, Instituto Oswaldo Cruz, Laboratório de Biologia, Controle e Vigilância de Insetos Vetores, Rio de Janeiro, RJ, Brasil; 2 Fundação Oswaldo Cruz-Fiocruz, Instituto René Rachou, Belo Horizonte, MG, Brasil; 3 Ministério da Saúde, Brasília, DF, Brasil; 4 Superintendência de Controle de Endemias, Laboratório de Entomologia Aplicada, Marília, SP, Brasil; 5 Université de Montpellier, Centre National de la Recherche Scientifique, Institut de Recherche pour le Développement, Maladies Infectieuses et Vecteurs: Écologie, Génétique, Évolution et Contrôle, Montpellier, France; 6 Universidade Federal do Rio de Janeiro, Instituto Nacional de Ciência em Entomologia Molecular, Rio de Janeiro, RJ, Brasil

**Keywords:** Aedes aegypti, vector control, insecticide resistance, Spinosad, Cielo, Fludora Fusion

## Abstract

**BACKGROUND:**

Insecticides play a critical role in controlling insect vectors, particularly during epidemics. Effective chemical control relies on the robust monitoring of insecticide resistance to guide evidence-based decision-making in vector control strategies.

**OBJECTIVES:**

This study assessed the susceptibility of *Aedes aegypti*, the primary vector of dengue, Zika, and Chikungunya viruses, to various larvicides and adulticides deployed during Brazil’s national campaigns from 2020 to 2023.

**METHODS:**

Mosquito collection was performed in 46 Brazilian municipalities using ovitraps. Eggs were transported to FIOCRUZ to establish the F1 and F2 generations. The Rockefeller strain was employed to determine the discriminating concentrations (DC) for the larvicide Natular™ 20EC (spinosad) and the adulticides Cielo™ (imidacloprid and prallethrin) and Fludora^®^ Fusion (clothianidin and deltamethrin) using a modified World Health Organization (WHO) bottle bioassay. These DCs were then used to estimate the resistance status of *Ae. aegypti* populations in the tested formulations. Resistance intensity was assessed by exposing mosquitoes to five, 10, or 20 times the DC concentrations.

**FINDINGS:**

All *Ae. aegypti* populations were fully susceptible to larvicide spinosad. However, resistance to both adulticide formulations was detected based on WHO criteria (mortality rates < 90%). Intensity assays revealed high to very high resistance to combined adulticide products.

**MAIN CONCLUSIONS:**

Our findings indicate the full susceptibility of *Ae. aegypti* populations in Brazil to spinosad, but substantial resistance to adulticides used in space spraying and residual applications, likely due to pre-existing pyrethroid resistance. However, the specific contributions of each active ingredient remain unclear, owing to the evaluation of the combined formulations. The efficacy of both traditional and alternative vector control strategies must be continuously evaluated and closely monitored to ensure the real-time assessment of their performance. For chemical control, future studies should prioritise the assessment of combination products in field trials, refining laboratory assays, and sustaining insecticide resistance surveillance to optimise control efforts in Brazil.

The mosquito *Aedes aegypti* (Linnaeus, 1762) is a dipteran insect capable of transmitting several etiological agents to humans, including arboviruses such as dengue (DENV), Zika (ZIKV) and Chikungunya (CHIKV) viruses.^1^ ^,^ ^2^ ^,^ ^3^ In 2023, the Americas reported over 4.2 million DENV cases^4^ with Brazil accounting for approximately 69% of this burden.^4^ By September 2024, Brazil had reported over 6 million probable DENV cases, highlighting the increasing public health challenge posed by this vector.^5^

Significant efforts have been made to mitigate the incidence of these arboviruses in Brazil. These include investments in diagnostic supplies, insecticides, and other control materials; research into novel vector control technologies; development of contingency plans for outbreak response; mobilisation of medical and vector control resources, training of health professionals; intersectoral collaborations; public information campaigns; social mobilisation, and, more recently, vaccination against DENV in a specific population range.^6^ ^,^ ^7^ Despite these efforts, recommended strategies have not been sufficiently effective to curb the proliferation of *Ae. aegypti* in most of the Brazilian territory, leading to intense virus circulation and recurrent outbreaks.^8^

Physical and mechanical measures, coupled with educational campaigns targeting the elimination of potential larval habitats, remain fundamental in vector control. The national programme also advocates systematic use of larvicides in permanent water storage containers and the application of adulticides during outbreaks.^9^ Additionally, widespread use of pyrethroid-based household products, such as aerosols for personal protection, contributes to environmental pyrethroid pressure.^10^ The extensive reliance on pyrethroids has driven the selection of highly resistant *Ae. aegypti* populations in Brazil, a trend that poses a significant threat to vector control both nationally and globally.^11^

Given these challenges, monitoring insecticide resistance has become essential to prevent failures in vector control programs. In Brazil, tests based on discriminating concentrations (DC) of insecticides have been used for over 20 years to classify mosquito populations as susceptible or resistant, guiding operational decisions.^12^ This approach represents a critical first step in implementing integrated resistance management and is often complemented by biochemical and molecular assays to identify resistance mechanisms.^13^ ^,^ ^14^ Recent updates to World Health Organization (WHO) guidelines introduced new DCs for key insecticide active ingredients and recommended WHO bottle bioassays to evaluate mosquito resistance to novel compounds unsuitable for filter paper impregnation.^15^ ^,^ ^16^ ^,^ ^17^

The Brazilian Ministry of Health (MoH) has monitored *Ae. aegypti* insecticide resistance since 1999, following WHO recommendations or adaptations thereof.^18^ During the 2017-2018 round of evaluations, resistance to the organophosphate malathion was identified in most populations, while incipient resistance to the IGR larvicide pyriproxyfen detected in the country’s Northeast region.^19^ Pyrethroid resistance-associated *kdr* mutations (Val410Leu, Val1016Ile and Phe1534Cys) have also been reported at high frequencies in northern and southern Brazil.^20^ In response to these findings, the MoH adopted new products: the larvicide Natular^TM^ 20EC and the adulticides Cielo^TM^ and Fludora^®^ Fusion, replacing previous formulations.^21^ ^,^ ^22^ ^,^ ^23^

Natular^TM^ 20EC contains spinosad, an active ingredient and a product of the aerobic fermentation of the soil actinobacterium *Sacharopolyspora spinosa*, belonging to the spinosyn class. Spinosad disrupts nicotinic acetylcholine receptors, making it effective as larvicide.^24^ ^,^ ^25^ Cielo^TM^ combines the neonicotinoid imidacloprid and pyrethroid prallethrin and is applied as a space spray. Fludora^®^ Fusion, a residual action formulation applied to wall surfaces, combines neonicotinoid clothianidin with the pyrethroid deltamethrin. Clothianidin and imidacloprid target nicotinic acetylcholine receptors, while deltamethrin and prallethrin act on voltage-gated sodium channels (Na_V_).^26^ Studies have demonstrated the efficacy of these combined formulations against various *Anopheles species* ^27^ ^,^ ^28^ ^,^ ^29^ ^,^ ^30^ and *Ae. aegypti*,^31^ ^,^ ^32^ particularly in areas with pyrethroid resistance, thereby supporting their adoption in Brazil.

As part of a large-scale insecticide resistance monitoring program, we evaluated the susceptibility of *Ae. aegypti* populations in Brazil to the larvicide Natular^TM^ 20EC and the adulticides Cielo^TM^ and Fludora^®^ Fusion. Using the WHO bottle bioassay, we determined the DCs for each formulation with the susceptible Rockefeller strain, and then applied these DCs to assess the presence and intensity of insecticide resistance in *Ae. aegypti* populations from 46 municipalities of Brazil. The findings of this study aim to support MoH optimisation of Brazil’s chemical vector control strategies for DENV prevention.

## MATERIALS AND METHODS

**Fig. 1:**
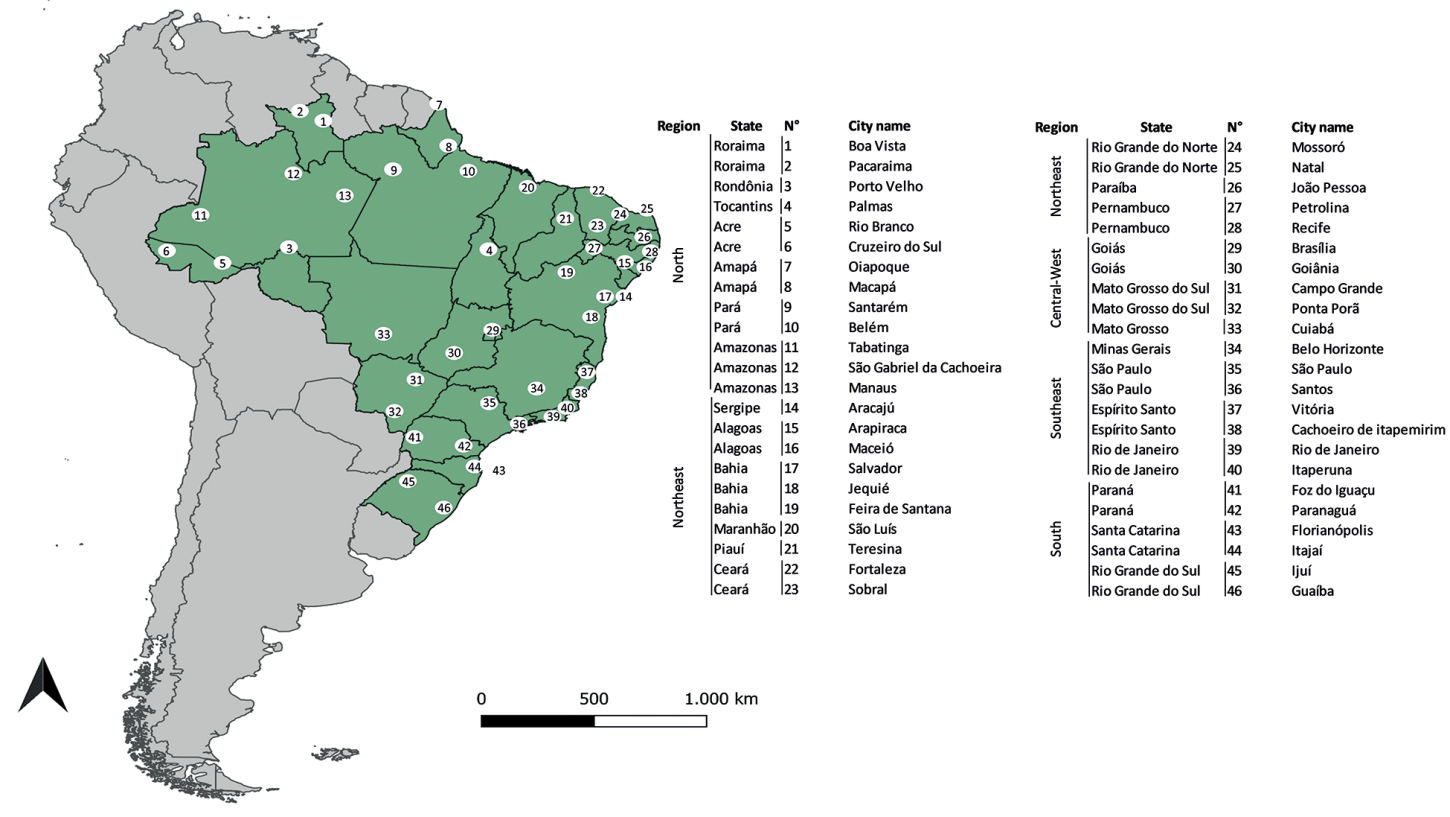
*Aedes aegypti* populations were assessed for insecticide resistance during the 2021-2023 campaign. The map shows Brazil (green) with state outlines. The municipalities where egg collections were performed are marked with number points, corresponding to the detailed list provided in the accompanying table.

*Selection of sites and training of field agents* - Collaborative efforts were undertaken for the 2020-2023 insecticide resistance monitoring (IRM) round in Brazil. The MoH selected 46 municipalities based on specific criteria, including state capital, international borders, key port areas, regions with high DENV incidence from 2015 to 2019, and areas with significant human mobility (Fig. 1). To ensure the success of mosquito collection, a tripartite agreement was established between the National Council of Health Secretaries (CONASS), the National Council of Municipal Health Secretaries (CONASEMS), and the MoH.

The collection methodology was introduced during two virtual events, which involved representatives from the 26 states, the Federal District, state capitals municipalities, international border areas, and the agencies CONASS, CONASEMS, and MoH. Local field agents received technical training in the IRM protocol, which included the installation of ovitraps, the proper removal of pallets with collected eggs, and the transport of samples to laboratories. The training sessions featured step-by-step instructions and a video tutorial to ensure consistency across all sites.^33^ During the training, the agents demonstrated a clear understanding and high level of engagement, as evidenced by their pertinent questions about ovitrap installation, drying process, egg counting, and packaging of pallets. Although no formal evaluation of the learning process was conducted, informal feedback further reinforces this observation. The participants were tasked with collecting and counting *Ae. aegypti* eggs, and shipping both the data and biological samples to the FIOCRUZ laboratory for further processing and analysis.

**Fig. 2:**
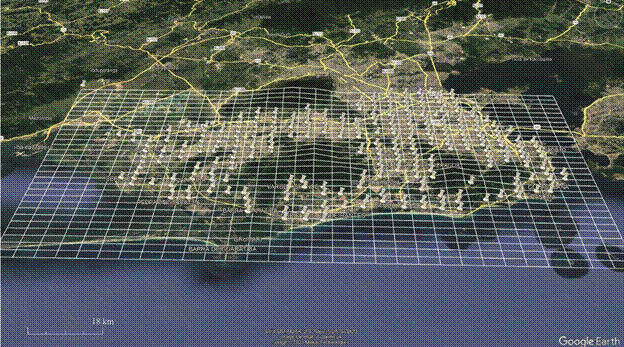
example of a grid created using Google Earth Pro to guide municipalities in ovitraps installation. The map depicts the city of Rio de Janeiro with grid cells measuring 1 km in height and 2 km in width.

*Mosquito collection* - Mosquito egg collections were conducted between January 2021 and September 2022, based on seasonality and logistical considerations for each municipality. Field agents from the Municipal Health Secretaries deployed ovitraps^33^ during routine entomological surveys following MoH recommendations^34^ and the training provided. The number of traps deployed was proportional to municipality size: 100 traps for municipalities with up to 50,000 houses, 150 traps for municipalities with up to 200,000 houses, 200 traps for municipalities with up to 500,000 houses, and 300 traps for municipalities with more than 500,000 houses. Ovitrap placement was determined using a grid system overlaid on municipal maps using the Google Earth software. The dimensions and number of squares were calculated by dividing the number of city blocks by the required number of ovitraps. Each grid square corresponds to the area where the agents placed a single ovitrap (Fig. 2).

After collection, samples were sent to the Laboratory of Biology, Control and Surveillance of Insect Vectors (LBCVIV) at IOC, FIOCRUZ, Rio de Janeiro (RJ), where each sample was coded to ensure data confidentiality. To assess phenotypic resistance in the field, *Ae. aegypti* population samples from 26 municipalities were analysed at LBCVIV, while 20 samples were forwarded to the Applied Entomology Laboratory (LEnA) at Superintendency of Endemic Disease Control (SUCEN), Marília, São Paulo (SP), to optimise the workload and reduce processing time.

*Egg hatching and rearing* - In the laboratories, egg palettes were submerged in water for three days to induce hatching. Groups of approximately 500 larvae were transferred to plastic trays (33 cm × 24 cm × 8 cm) containing 1 L of dechlorinated water. For each mosquito population, a maximum of 5,000 larvae (10 trays of 500 larvae each) were reared. Every three days, 450 mg of Tetra Marine^®^ Granules fish food (Tetra, Blacksburg, VA, USA) was added to each tray until the larvae reached the pupal stage.

Pupae were transferred to cylindrical cardboard entomological cages (16 cm diameter x 18 cm height) for adult emergence. Within 48 h of emergence, *Ae. aegypti* adults were visually identified and transferred to new cages with a maximum of 500 females and 500 males per cage. The adults had continuous access to 10% sucrose solution.

Females were blood-fed three days after emergence using a Hemotek^®^ artificial feeder (Discovery Workshops, Accrington, UK) with citrated rabbit blood. Dark cups containing water and lined with filter paper were provided for oviposition three-four days post-blood feeding. This procedure was repeated as required for up to three generations. Populations with fewer than 100 females in the F1 generation were discarded and additional collections were performed. The insectary conditions were maintained at 26 ± 2ºC, 70 ± 15% relative humidity and 12:12-h light/dark photoperiod. Water from the local supply was kept in the insectarium in an open, screened drum and aerated with an aquarium pump for at least 2 h to allow dechlorination before being used to hatch the eggs and maintain the larvae. Filtered water from a local supply was used for conducting the bioassays.

*Mosquito colony used for determining discriminating concentrations* - The susceptible *Ae. aegypti* Rockefeller strain was used to determine the DCs for each insecticide formulation. This strain (*Aedes aegypti* ROCK MRA-734) was obtained from the BEI Resources consortium, supported by the National Institute of Allergy and Infectious Diseases (NIAID), National Institute of Health (NIH), USA. This Rockefeller strain has been maintained at LBCVIV laboratory since 2020.^35^

*Insecticides* - The larvicide spinosad was provided by the MoH as a liquid emulsifiable concentrate (Natular^TM^ 20EC) containing 20.6% spinosad active ingredient (a.i.) (Clarke Mosquito Control Products, Inc., St. Charles - USA). Following WHO guidelines, a stock solution of 3,000 mg/L was prepared by diluting Natular^TM^ 20EC in ethanol for use in bioassays.^36^ Cielo^TM^ (Clarke Mosquito Control Products, St. Charles, IL, USA) and Fludora^®^ Fusion (Envu, Monheim, Germany) were provided by the respective manufacturers. Cielo^TM^ ULV contains 30 g/kg (3% w/w) of neonicotinoid imidacloprid and 7.5 g/kg (0.75% w/w) of the pyrethroid prallethrin. Fludora^®^ Fusion contains 500 g/kg (50%) of the neonicotinoid clothianidin and 62.5 g/kg (6,25%) of the pyrethroid deltamethrin. Stock solutions of Cielo^TM^ (260 mg/L) and Fludora^®^ Fusion (500 mg/L) were prepared in acetone for the bioassays.

*Determination of DCs: Natular* ^*TM*^ *20EC against susceptible Ae. aegypti* - Concentration-response bioassays were conducted to estimate lethal concentrations (LC_50_ and LC_99_) and determine the tentative DCs for Natular^TM^ 20EC using third-instar larvae or the Rockefeller *Ae. aegypti* strain. Ten concentrations of Natular^TM^ 20EC (0.075-0.65 mg/L) were tested, prepared by diluting the stock solution in ethanol and water. Each concentration was tested in four replicates, with 20 larvae per disposable plastic cup containing 100 mL of solution. Controls consisted of cups without insecticide and containing ethanol at the highest concentration (0.6%). Mortality was recorded 24 h post-exposure, as established by Dias et al.^25^ Four independent bioassays were performed on different days using larvae from distinct egg batches. These trials, conducted to determine the discriminating concentrations of spinosad, were performed simultaneously at LBCVIV and LEnA, with both laboratories employing a double-anonymous approach under controlled environmental conditions (temperature of 26 ± 2ºC and relative humidity of 70 ± 15%). We used the mortality rates at 24 h post-exposure to calculate the LCs using Probit analysis^37^ with the POLO-PC software (Leora Software, Berkeley, CA). The DC was defined as double the LC_99_ value, as per WHO guidelines.^15^

*Cielo* ^*TM*^ *and Fludora* ^®^ *Fusion against Ae. aegypti* - At the time of the study, no WHO-recommended DCs or testing protocols were available for some active ingredients (*e.g.*, clothianidin, imidacloprid, and prallethrin). Since these formulations are mixtures containing active ingredients and other inert substances that enhance insecticide solubility, stability, absorption, and efficacy,^38^ ^,^ ^39^ resistance was assessed using ready-to-use products with the WHO bottle bioassay method.^15^ ^,^ ^16^

Bottle coating and bioassays were conducted according to WHO protocols.^15^ ^,^ ^40^ Bottles were impregnated with 10 concentrations of each product (Cielo^TM^: 0.8 - 3 µg/mL; Fludora^®^ Fusion: 0.12 - 2 µg/mL) and stored horizontally, protected from light, under controlled conditions (temperature of 26 ± 2ºC and 70 ± 15% RH). Rockefeller strain mosquitoes were exposed to treated bottles for 1 h, transferred to holding cages, and provided with 10% sucrose solution. Mortality was recorded at 24 and 48 h. Four independent bioassays were conducted for each product on different days, using mosquitoes from distinct egg batches. DCs were calculated by Probit analysis.

*Phenotypic resistance of field Ae. aegypti populations*: *resistance to spinosad Natular* ^*TM*^ *20EC* - Using the DC determined with Rockefeller mosquitoes, field populations were tested for resistance to Natular^TM^ 20EC as per WHO guidelines.^36^ One test consisted of 160 third-instar larvae of each population exposed to DC in eight plastic cups (20 larvae/cup, 250 mL solution). Controls included four cups containing 0.4% ethanol solution (solvent control) and four cups with Rockefeller larvae exposed to DC (positive control). Mortality was recorded 24 h post- exposure, and Abbott’s formula was applied when control mortality ranged from 5 to 20%.^41^ If pupation occurred in any cup or if mortality was greater than 20% in the control group, the test was discarded and repeated.

For each population, we conducted four independent tests on different days with larvae from different batches of eggs from the same generation. The environmental conditions were maintained at 26 ± 2ºC and 70 ± 15% RH. The populations were classified as follows:^16^ ^,^ ^36^

Susceptible: > 98% mortality.

Possible resistance: 90-98% mortality.

Resistance: < 90% mortality.

*Resistance to Cielo* ^*TM*^ *and Fludora* ^®^ *Fusion* - Field populations were tested using WHO bottle bioassays. Groups of 20-25 females (three-five days old, unfed with blood) were exposed to four bottles coated with DC of each insecticide (80-100 females/ test). Controls included two bottles treated with acetone (negative control) and two bottles treated with insecticide DC using Rockefeller females (positive control). After 1 h of exposure, knockdown rates were recorded, and surviving mosquitoes were transferred to cages containing 10% sucrose solution. Mortality was recorded at 24 h, and Abbott’s formula was applied for control mortality between 3% and 10%, while tests with control mortality > 10% or Rockefeller mortality < 100% were discarded and repeated.^41^ The Mortality rate of each population was considered the average of the four different assays, each using different batches of mosquitoes. During the entire evaluation period, the temperature and humidity were controlled (26 ± 2ºC and 70 ± 15%, respectively).

Populations with < 98% mortality were subjected to intensity assays using concentrations of 5x, 10x, and 20x × DC. Mortality was interpreted according to the WHO intensity criteria (Table).

**TABLE:** Classification of resistance intensity in *Aedes aegypti* populations based on the average mortality rate recorded 24 h after 1-hour exposure to the insecticide

Resistance intensity	Mortality criteria
-	Mortality > 98% in the DC (susceptible)
+	Mortality < 98% in the DC and > 98% in 5x DC
++	Mortality < 98% in 5x DC and > 98% in 10x DC
+++	Mortality < 98% in 10x DC and > 98% in 20x DC
++++	Mortality < 98% in 20x DC

DC: discriminating concentration.

*Ethics* - No ethical licenses or permits were required for the use of *Aedes* collection in Brazil. Females were fed citrated rabbit blood using a Hemotek^®^ artificial feeder, under the studies codes L-004/2018 and L-004/2018-A1, approved by the IOC Ethics Committee for Animal Use (CEUA IOC, FIOCRUZ).

## RESULTS

**Fig. 3:**
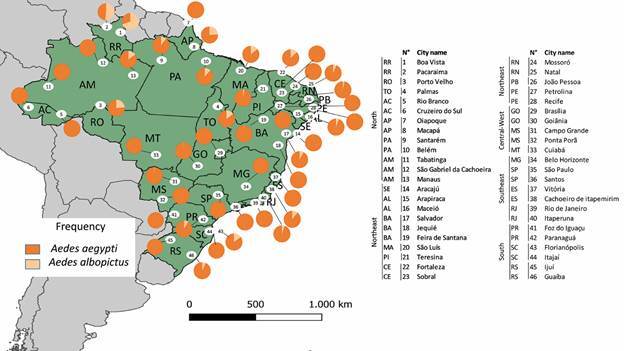
relative proportions of *Aedes aegypti* and *Aedes albopictus* in 46 municipalities across Brazil during the 2021-2023 insecticide resistance monitoring campaign. The map illustrates the localities identified by numbered markers in the legend, arranged according to their geographical coordinates, and categorised within Brazil’s five macro-regions.

*Baseline information* - A total of 524,615 eggs were collected across the 46 municipalities included in the study, and 105,604 mosquitoes were identified at the species level. Detailed information regarding collection sites, dates, number of ovitraps deployed, and total number of adult mosquitoes (separated by sex and species) is provided in Supplementary data (Table). *Ae. aegypti* was identified in the 46 municipalities, being the only species in 13 of them: Cruzeiro do Sul and Rio Branco (AC), São Gabriel da Cachoeira, and Tabatinga (AM), Oiapoque (AP), Sobral (CE), Mossoró (RN), Petrolina (PE), Belo Horizonte (MG), São Paulo (SP), Foz do Iguaçu (PR), Ijuí (RS), and Cuiabá (MT). It was found at higher frequencies than *Aedes albopictus* in an additional 31 municipalities (Fig. 3). Conversely, *Ae. albopictus* was absent in 13 of the 46 municipalities evaluated. Among the 33 municipalities in which *Ae. albopictus* was found, the Northern region exhibited the highest abundance, Boa Vista (69,7%) and Pacaraima (51,7%), while the Central-West region had the lowest abundance.

**Fig. 4:**
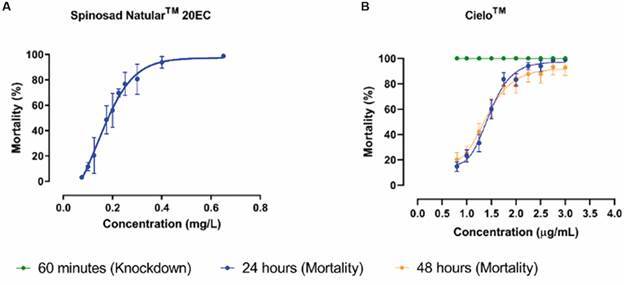
concentration-response curves for the evaluated insecticides against *Aedes aegypti* Rockefeller strain. Panel A: concentration-response curve of third-instar larvae exposed to Natular™ 20EC. Panel B: concentration-response curve for the adulticide Cielo™.

*Resistance of Ae. aegypti from Brazil to spinosad (Natular* ^*TM*^ *20EC)* - Larval bioassays determined the LC_99_ for the susceptible Rockefeller strain exposed to Natular^TM^ 20EC to be 0.6 mg/mL, as illustrated in the concentration-response curve (Fig. 4A). Consequently, the DC for spinosad was established as 1.2 mg/L, which was subsequently employed to evaluate the resistance of the field-collected *Ae. aegypti* populations.

All 46 *Ae. aegypti* populations tested demonstrated full susceptibility to Natular^TM^ 20EC, with 24-h mortality rates exceeding 98%, in accordance with the WHO criteria.

*Resistance of Ae. aegypti from Brazil to Cielo* ^*TM*^ *and Fludora* ^®^ *Fusion*: d*etermination of DCs* - For the susceptible Rockefeller strain, the LC_99_ values were determined as 3 µg/mL for Cielo^TM^ (Fig. 4, Panel B) and 1.25 µg/mL for Fludora^®^ Fusion. A DC of 6 µg/mL was established for Cielo^TM^. For resistance intensity evaluation, concentrations of 30, 60, and 120 µg/mL were used, corresponding to 5x, 10x and 20x × DC, respectively. For the Fludora^®^ Fusion, we obtained divergent results between LBCVIV (DC 2.5 µg/mL) and LEnA (DC 10 µg/mL). After further verification, we adopted a DC of 10 µg/mL, as it has the potential to provide a clearer distinction of the resistance status in mosquito populations, and the resistance intensity evaluations considered concentrations of 50, 100, and 200 µg/mL, corresponding to 5x, 10x, and 20x × DC.

*Field population resistance* - Preliminary tests revealed that mortality in field populations was consistently below 98% when exposed to DCs of both Cielo^TM^ and Fludora^®^ Fusion. Consequently, evaluations began at 5x DC or higher.

**Fig. 5:**
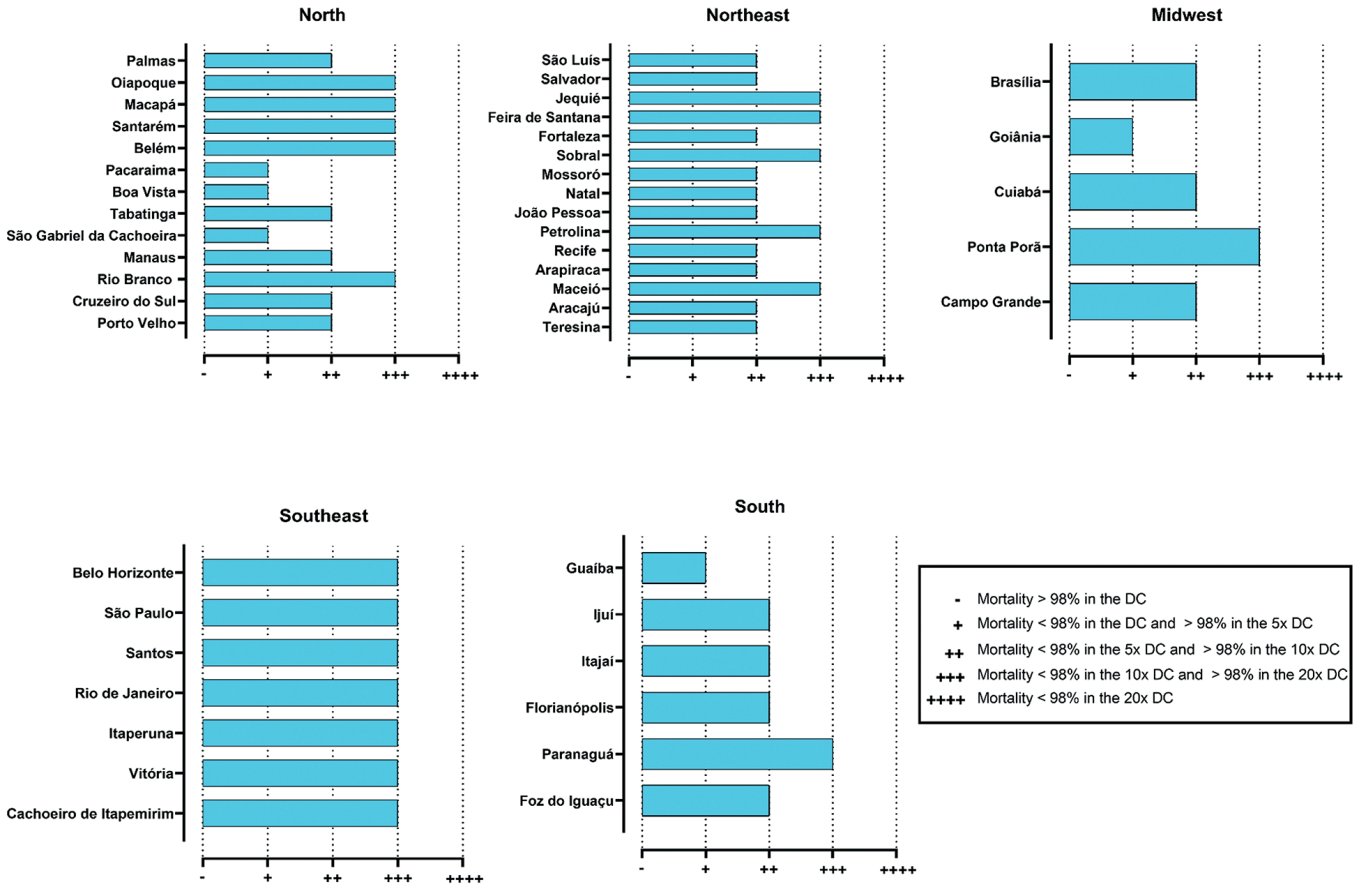
phenotypic resistance of *Aedes aegypti* exposed to the discriminating concentration (DC) of Cielo^TM^ across different regions of Brazil. Resistance was classified based on the average mortality rates of mosquitoes 24 h after 1-hour exposure to DC (6**ºµg/mL of** Cielo^TM^) and multiple-fold concentrations of DC (5x, 10x, and 20x).

**Fig. 6:**
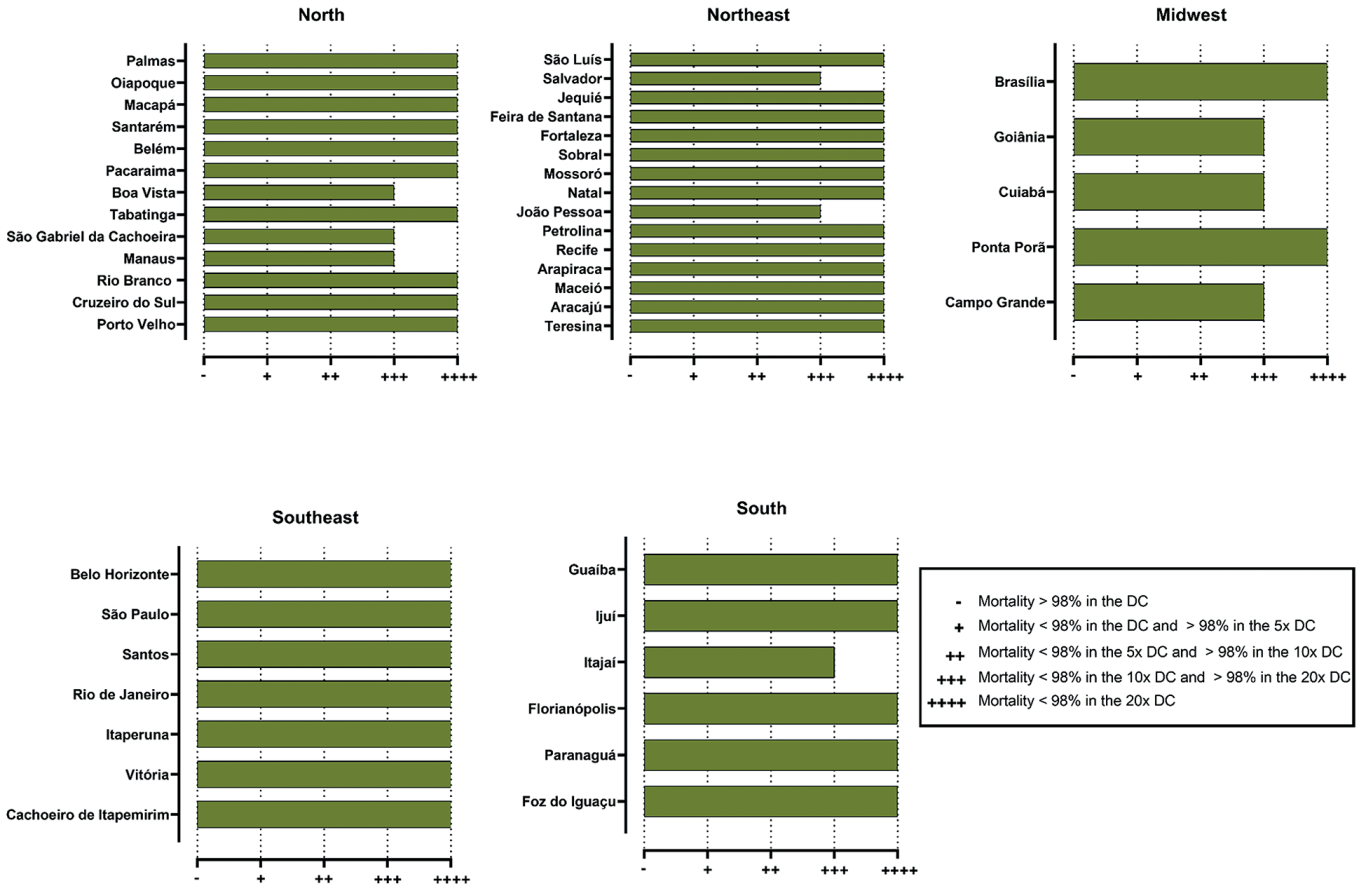
phenotypic resistance of *Aedes aegypti* exposed to a discriminating concentration (DC) of Fludora^®^ fusion across different regions of Brazil. Resistance was classified based on the average mortality rates of mosquitoes 24 h after 1-hour exposure to the DC (10 µg/mL of Fludora^®^ Fusion) and multiple-fold concentrations of the DC (5x, 10x, and 20x)*.*

According to the WHO criteria,^40^ all *Ae. aegypti* populations tested demonstrated resistance to both the Cielo^TM^ and Fludora^®^ fusion. Mortality rates after 1-h exposure to 5x DC followed by 24-h observation were < 98%, indicating moderate to high resistance. High levels of resistance were observed across several Brazilian regions (Figs 5-6).

*Regional variation in resistance to Cielo™* - The levels of resistance to Cielo™ varied by region. The Southeast region was the most concerning, where 100% of the tested populations exhibited high resistance (+++). In the Northeast region, there was a balanced distribution of moderate (++) and high (+++) resistance. The Northern, Central-West, and Southern regions ranged from low (+) to high (+++) resistance to Cielo™.

*Regional variation in resistance to Fludora* ^®^ *Fusion* - *Aedes aegypti* populations demonstrated high DC resistance in the Fludora^®^ Fusion (Fig. 6). Evaluation of resistance across Brazil revealed distinct regional patterns. The Southern region was predominantly characterised by very high resistance, with five of six populations evaluated demonstrating the maximum resistance level (++++). The Southeastern region was remarkably uniform, and all seven tested populations exhibited very high resistance (++++). In the Central-Western region, three populations showed high resistance (+++), while two displayed very high resistance (++++). The Northeastern region showed 13 of the 15 tested populations presenting very high resistance (++++). Finally, the Northern region showed a similar trend, with most populations exhibiting very high resistance (++++), and three populations (Boa Vista, São Gabriel da Cachoeira, and Manaus) showing high resistance (+++)

## DISCUSSION

This study evaluated the susceptibility to *Ae. aegypti* populations in Brazil to insecticides used for the control of DENV vectors from 2020 to 2023. Although all 46 mosquito populations exhibited full susceptibility to the larvicide spinosad (Natular^TM^ 20EC), high and widespread resistance to the adulticides Cielo^TM^ and Fludora^*®*^ Fusion was observed.

The presence of *Ae. albopictus* in Brazil was first recorded in 1980’s in Southeastern states, expanding toward the other regions in the next decades.^42^ We observed the presence of the Asian tiger mosquito in 33 (72%) localities, suggesting a geographic expansion, compared to 60% in the previous monitoring round.^19^ This trend can be clearly observed in some locations assessed in both studies- for example, in Boa Vista, where its presence increased from 0.1% to 70%, and in Palmas, where it rose from 4.9% to 15%. Additionally, in Macapá, where *Ae. albopictus* was not detected in the previous round, the current study recorded a presence exceeding 30%. Climate changes may be facilitating its dispersion and adaptation to other environments.^43^ For example, although *Ae. albopictus* had not been detected in Roraima State (North region) in a previous study (2016-2018), we found it in both Pacaraima and Boa Vista municipalities, yet under proportions higher than *Ae. aegypti*.^10^ The sympatric presence of *Ae. albopictus* and *Ae. aegypti* in certain areas mirrors trends documented elsewhere in the Americas.^44^ ^,^ ^45^ ^,^ ^46^ ^,^ ^47^ This expansion raises concerns about increasing exposure to *Ae. albopictus*, leading to potential selection pressures similar to those observed for *Ae. aegypti*. *Ae. albopictus* has demonstrated competence for several arboviruses, including CHIKV, yellow fever (YFV), ZIKV, and DENV, under laboratory conditions,^48^ ^,^ ^49^ ^,^ ^50^ ^,^ ^51^ and has been found naturally infected with ZIKV and DENV in Brazil.^52^ ^,^ ^53^ Monitoring its distribution and insecticide resistance status will be essential for vector control strategies, particularly if its role in arbovirus transmission under Brazilian environmental conditions is confirmed.^43^ ^,^ ^54^

Our findings confirm the full susceptibility of *Ae. aegypti* populations to Natular™ 20EC, consistent with previous studies.^25^ The bioassays and resistance criteria were adapted from WHO guidelines due to the absence of specific protocols for evaluating formulated products.^16^ ^,^ ^36^ The DC established for Natular™ 20EC was based on the Rockefeller strain and was used to assess field populations. Although the results are promising for larval control, continuous monitoring is essential for the detection of potential resistance development. Resistance to spinosad has been documented in other insect species^55^ and may arise in mosquitoes following prolonged exposure, as demonstrated in laboratory-selected populations over 15 generations.^56^ Spinosad remains a valuable tool in Brazil’s integrated vector management programs because of its efficacy, residual activity,^25^ relative toxicity,^57^ and lack of cross-resistance with other larvicides.

To ensure accuracy and reliability, DCs should be determined by more than one laboratory using the same reference strain in order to reduce the risk of overestimating or underestimating the resistance status of natural populations. Nevertheless, discrepancies between laboratories must be acknowledged. In our case, the determination of DC for Fludora^®^ Fusion yielded divergent results between the two laboratories (2.5 µg/mL vs. 10 µg/mL). Such variation, however, is common in multicentre studies, as recently reported in a WHO multicentre study.^15^ ^,^ ^58^ Despite differences in results with the Rockefeller strain, we opted for the higher dose, in accordance with WHO recommendations.^15^ This approach seemed appropriate, since we did not identify any population with mortality above 98%, even when using the highest concentration. In contrast Natular™ 20EC, high to very high levels of resistance to Cielo^TM^ and Fludora Fusion were observed in *Ae. aegypti* populations across Brazil, based on the WHO criteria.^40^ Our results showed that resistance to Cielo^TM^ was more heterogeneous, with the highest prevalence in the Southeastern region, whereas resistance to Fludora^*®*^ Fusion was more uniformly distributed, with the Southeastern and Southern regions exhibiting the highest levels. In general, considering the overall scenario, *Ae. aegypti* populations in Brazil appear to be less susceptible to Fludora^*®*^ than to Cielo™. Furthermore, resistance to Fludora^*®*^ Fusion tends to be more intense than resistance to Cielo™ across the country. Interpretation of these findings must be done cautiously because of the lack of international standards for evaluating resistance to formulated products or those containing multiple active ingredients. At the time of the study, WHO-recommended DCs were unavailable for imidacloprid, clothianidin, and prallethrin, necessitating the use of the final formulations for resistance testing. However, it is important to recognise that testing individual active ingredients does not account for potential interactions that may occur in formulated products, such as additive or synergistic effects. These interactions could influence the overall effectiveness and resistance patterns observed, highlighting the need for careful interpretation when using formulated products for resistance testing.

DC-combined formulations were significantly lower than the individual active ingredients. For example, the DC of Cielo^TM^ (6 *μg*/mL) contains 0.18 *μg* a.i./mL of imidacloprid and 0.045 *μg* ai/mL of prallethrin -approximately 600 times lower than the WHO-recommended DC for prallethrin alone (30 *μg*/mL) against *Ae. aegypti* in WHO bottle bioassay.^15^ Similarly, the DC for Fludora^*®*^ Fusion (10 *μg*/mL) contains 0.625 *μg* a.i./mL of deltamethrin and 5 *μg* a.i./mL of clothianidin - four times lower than the WHO-recommended DC for clothianidin against *Ae. aegypti*.^59^ This discrepancy suggests a potential synergistic effect between active ingredients with distinct models of action, as documented in other insecticide mixtures.^60^ ^,^ ^61^ However, due to the lack of DC assessments specifically addressing the combinations present in the adulticides tested in this study, there is a clear need for direct evaluation to verify the existence of a synergistic effect of the insecticides in these formulations.

In the case of Cielo™ and Fludora^*®*^ Fusion, it is difficult to isolate the effects of each active ingredient, making it unclear whether mosquito resistance is due to neonicotinoids, pyrethroids, or both. This ambiguity introduces potential bias, complicating the interpretation of the resistance intensity data. Moreover, the different modes of action and characteristics of these compounds may require alternative testing methods, as suggested recently by the WHO.^15^ Testing each insecticide separately is preferable to isolate the effects of each active ingredient and to minimise bias in future studies. This is now possible for some insecticides that already have a DC validated by the WHO, such as clothianidin, deltamethrin, and prallethrin. However, developing appropriate methodologies and norms to assess mosquito resistance to insecticide combinations is required, considering the increasing number of combined vector control products under development and assessment by the WHO PQT.

Finally, it is important to note that insecticide resistance does not necessarily equate to vector control failure. Field studies have demonstrated that the Cielo™ and Fludora^*®*^ Fusion remain effective against *Ae. aegypti* populations, even in areas with pyrethroid resistance. In ultra-low volume (ULV) applications, both susceptible and pyrethroid-resistant *Ae. aegypti* populations from Puerto Vallarta, Mexico, were classified as susceptible to Cielo™ after 24 h of exposure.^62^ Similarly, Fludora^*®*^ Fusion demonstrated high efficacy against pyrethroid-resistant malaria vectors in Africa, with residual activity lasting 7-10 months.^63^ Additional efficacy trials with clothianidin and imidacloprid formulations in the Americas are critical to support the Ministries of Health in evaluating the benefits of using combination products over single-insecticide options under different entomological settings. These data will aid in optimising vector control strategies to reduce the impact of outbreaks, while ensuring cost-effective resource use. Implementing integrated vector management remains the cornerstone for mitigating insecticide resistance^15^ and ensuring the long-term sustainability of vector control programs.
