# Supplementary material for: Susceptibility of Aedes aegypti to spinosad larvicide and space spray adulticides in Brazil
Source: Mem Inst Oswaldo Cruz. 2025 Jul 11;120:e240270. doi: 10.1590/0074-02760240270 (PMC12252664; doi:10.1590/0074-02760240270)
Supplement: Supplementary file 1 [file 1678-8060-mioc-120-e240270-s.pdf]

TABLE

Data on location, egg collection period, number of pallets installed and eggs, number of mosquitoes per species and sex screened to obtain specimens for insecticide resistance evaluation

									Identification						
				Collection					Mosquitoes	*Aedes aegypti*			*Aedes albopictus*		
Region	State	IBGE Code	Town	Collection period	Number of pallets	Positive pallets	Total Number of eggs	Average number of eggs per pallets	Total number	♂	♀	%	♂	♀	%
N	RO	110020	Porto Velho	November 4th to 21st, 2021	300	215	17.139	80	4246	1727	1892	85.2	334	293	14.8
N	AC	120020	Cruzeiro do Sul	May 09th to 24th, 2021	74	NA	NA	NA	883	455	428	100	0	0	0
N	AC	120040	Rio Branco	May 18th to 31st, 2021	138	81	4.447	55	3166	1391	1775	100	0	0	0
N	AM	130260	Manaus	September 20th to 28th, 2021	600	110	4.110	37	1127	427	529	84.8	76	95	15.2
N	AM	130380	São Gabriel da Cachoeira	March 17th to 28th, 2022	98	68	3.647	54	270	156	114	100	0	0	0
N	AM	130406	Tabatinga	September 29th to October 11th, 2021	200	66	4.455	68	1359	711	648	100	0	0	0
N	RR	140010	Boa Vista	October 27th to November 11th, 2021	300	156	7.083	45	2729	419	409	30.3	870	1031	69.7
N	RR	140045	Pacaraima	October 13th to 19th, 2021	200	72	3.318	46	551	127	139	48.3	118	167	51.7
N	PA	150140	Belém	NA	NA	NA	NA	NA	3461	1549	1508	88.3	126	278	11.7
N	PA	150680	Santarém	December 3rd to 15th, 2021	300	210	24.822	118	1760	758	862	92.0	53	87	8.0
N	AP	160030	Macapá	NA	NA	NA	NA	NA	2209	766	747	68.5	315	381	31.5
N	AP	160050	Oiapoque	NA	NA	23	NA	NA	198	109	89	100	0	0	0
N	TO	172100	Palmas	January 11th to 25th, 2021	290	70	7.172	102	3098	1371	1264	85.1	183	280	14.9
NE	MA	211130	São Luís	September 13th to 26th, 2022	400	230	17.533	76	2372	779	1198	83.3	101	294	16.7
NE	PI	221100	Teresina	September 27th to October 14th, 2021	413	215	10.335	48	2208	1066	1098	98.0	12	32	2.0
NE	CE	230440	Fortaleza	April 16th to May 3rd, 2021	596	355	31.923	90	4206	1629	1946	85.0	249	382	15.0
NE	CE	231290	Sobral	March 29th to April 16th, 2021	300	107	8.604	80	425	185	240	100	0	0	0
NE	RN	240800	Mossoró	February 23rd to March 16th, 2021	300	132	16.640	126	243	110	133	100	0	0	0
NE	RN	240810	Natal	February 7th to 25th, 2022	472	268	23.294	87	4258	2000	2007	94.1	105	146	5.9
NE	PB	250750	João Pessoa	January 25th to February 12th 2021	400	268	14.877	56	4731	2281	2329	97.4	54	67	2.6
NE	PE	261110	Petrolina	February 22nd to March 12th 2021	293	101	3.575	35	568	277	291	100	0	0	0
NE	PE	261160	Recife	February 22nd to March 11th 2021	660	457	47.881	105	4998	2473	2500	99.5	8	17	0.5
NE	AL	270030	Arapiraca	February 24th to March 8th 2021	338	115	6.479	56	2947	1430	1508	99.7	2	7	0.3
NE	AL	270430	Maceió	March 10th to 23rd, 2021	398	172	10.629	62	1937	909	918	94.3	57	53	5.7
NE	SER	280030	Aracaju	March 9th to 26th, 2021	347	187	19.457	104	5013	2500	2500	99.7	6	7	0.3
NE	BA	291080	Feira de Santana	April 7th to 29th, 2021	288	84	4.107	49	1454	711	736	99.5	6	1	0.5

				Collection					Identification						
									Mosquitoes	*Aedes aegypti*			*Aedes albopictus*		
Region	State	IBGE Code	Town	Collection period	Number of pallets	Positive pallets	Total Number of eggs	Average number of eggs per pallets	Total number	♂	♀	%	♂	♀	%
NE	BA	291800	Jequié	June 11th to July 6th 2021	240	107	6.200	58	1410	702	705	99.8	2	1	0.2
NE	BA	292740	Salvador	April 26th to May 10th 2021	697	319	23.520	74	4394	2047	1991	91.9	201	155	8.1
SE	MG	310620	Belo Horizonte	April 11th to 22nd 2022	1.138	476	46.064	97	5012	2509	2503	100	0	0	0
SE	ES	320120	Cachoeiro do Itapemirim	May 11th to June 2nd 2021	198	112	7.290	65	2109	956	930	89.4	156	67	10.6
SE	ES	320530	Vitória	December 6th to 20th, 2021	292	223	31.934	143	4258	2000	2007	94.1	105	146	5.9
SE	RJ	330220	Itaperuna	March 1st to May 20th, 2021	180	102	6.735	66	1349	640	641	95.0	22	46	5.0
SE	RJ	330455	Rio de Janeiro	May 7th to 25th, 2021	590	318	14.891	47	2478	1090	1104	88.5	135	149	11.5
SE	SP	354850	Santos	February 22nd to March 5th 2021	330	261	28.000	107	2833	1415	1416	99.9	0	2	0.1
SE	SP	355030	São Paulo	May 17th to 26th, 2021	600	52	1.523	29	635	338	297	100	0	0	0
S	PR	410830	Foz do Iguaçu	February 17th to March 4th 2021	300	257	24.505	95	3873	2019	1854	100	0	0	0
S	PR	411820	Paranaguá	April 20th to May 4th 2021	200	85	3.671	43	1670	807	715	91.1	81	67	8.9
S	SC	420540	Florianópolis	April 26th to May 14th 2021	402	85	2.139	25	558	249	220	84.1	39	50	15.9
S	SC	420820	Itajaí	May 3rd to 18th, 2021	288	NA	NA	NA	1036	608	397	97.0	19	12	3.0
S	RS	431020	Ijuí	March 5th to April 12th 2021	200	139	7.209	52	1209	701	508	100	0	0	0
S	RS	430930	Guaíba	January 12th to 24th, 2022	200	NA	NA	NA	1674	730	835	93.5	57	52	6.5
CW	MS	500270	Campo Grande	February 22nd to March 10th 2021	384	240	7.647	32	2452	1117	1312	99.1	11	12	0.9
CW	MS	500660	Ponta Porã	March 24th - April 5th 2021	200	157	6.642	42	1257	641	615	99.9	0	1	0.1
CW	MT	510340	Cuiabá	July 12th to 19th, 2021	400	37	1.002	27	743	349	394	100	0	0	0
CW	GO	520870	Goiânia	April 26th to May 11th 2021	301	149	10.674	72	4307	2274	2010	99.5	15	8	0.5
CW	DF	530010	Brasília	November 18th to December 3rd 2021	446	68	3.442	51	1930	947	982	99.9	1	0	0.1

N: North; NE: Northeast; SE: Southeast; S: South; CW: Central-West; RO: Rondônia; AC: Acre; AM: Amazonas; RR: Roraima; PA: Pará; AP: Amapá; TO: Tocantins; MA: Maranhão; PI: Piauí; CE: Ceará; RN: Rio Grande do Norte; PB: Paraíba; PE: Pernambuco; AL: Alagoas; SER: Sergipe; BA: Bahia; MG: Minas Gerais; ES: Espírito Santo; RJ: Rio de Janeiro; SP: São Paulo; PR: Paraná; SC: Santa Catarina; RS: Rio Grande do Sul; MS: Mato Grosso do Sul; MT: Mato Grosso; GO: Goiás; DF: Distrito Federal; NA: Not Available; ♂: male; ♀: female; IBGE Code: a number assigned by the Brazilian Institute of Geography and Statistics to identify different geographical units within Brazil.
